# Hospital and Institutional News

**Published:** 1913-08-30

**Authors:** 


### HOSPITAL AND INSTITUTIONAL NEWS.

### "THE HOSPITAL'S" SPECIAL EDUCATIONAL NUMBER.

Next week, in our issue dated September 6, we shall publish a Special Educational Number of The Hospital, which will comprise not merely an account of the curricula of the various Medical Faculties likely to be helpful to the student at the opening of his profession, but also a series of articles describing the various branches of the medical profession now open to medical men. As we explained last week, these openings have increased very largely of late years—so largely, in fact, that information concerning what may be called the main professions of medicine, such as the Army and Navy Medical Services, the Colonial Services, and the Public Health branches, is now perhaps almost unattractive. It is to the newer branches of medicine that the medical student and the newly qualified practitioner are turning their eager attention. This being so, The Hospital's Educational Number will include special articles upon the new appointments under the recently organised "Tuberculosis Service"; the "Prospects of Panel Practice"; the advantages and disadvantages of the "Hospital Branch of the Poor-Law Medical Service"; "Medical Journalism," an important bypath of medicine to which a certain class of man is attracted, and about which authoritative information is hard to come by. We hope also to include—last, but by no means least—an article dealing with the "Scarcity of Resident Medical Officers," which explains the present position of affairs, how it has been brought about, and how it may be remedied, for which a special inquiry was made in order to cover the whole ground of a difficult and locally variable problem. This article should prove extremely valuable to institutional managers, for it explains to them how closely their interests are bound up with the medical schools, and in what respects the hospitals themselves are responsible for the present difficulty experienced by them in filling resident appointments. The Special Educational Number will be issued at the ordinary price of The Hospital—namely, one penny.

### AN INFIRMARY SUPERINTENDENT AS SANITARY INSPECTOR.

A startling report from Dr. W. H. Bruce, medical superintendent of Southwark Infirmary, was read last week at a meeting of the Southwark Board of Guardians, in which he said that there had been eighteen cases of infective enteritis among the child patients, and that four had died. He attributed the cause of the outbreak entirely to the poisoning of the children by the refuse on an adjoining railway siding, which the Camberwell Borough Council was having removed. As a result of communications with the Local Government Board the Camberwell Borough Council has placed screens on the railway trucks to prevent the dust from being blown about. Mr. E. H. Norman emphasised the importance of immediate steps being taken to abate the nuisance, but the Chairman of the Southwark Guardians, Mr. J. Osborn, said that abatement was not sufficient. The nuisance must be stopped completely, and with his support a deputation was appointed to wait on the Local Government Board before resorting to legal action. The screens have rightly been described by Dr. Bruce as a farce, and, we fear, could only have been put forward as an excuse for doing nothing. Uncovered refuse must not be allowed to remain in any place where it is likely to be a source of infection. In other words, it must not be allowed to remain in unprotected heaps anywhere. Nothing short of that will be of any real value. Dr. Bruce and Mr. Osborn are to be congratulated on the powers of diagnosis and executive promptitude which they have shown respectively.

### THE POPULARITY OF ARTESIAN WELLS.

A feeling of impatience is generally felt by Londoners when they are told by visitors that their water is probably the purest of any great city in the world. The engineering feat of the New River Company and the river's source we feel to be more deserving of praise. The Medical Officer of Health for the City, however, is providing a more local reason for the satisfaction of Londoners at their water-supply, when he draws attention to the increasing number of buildings, hospitals among them, which are deriving their water from artesian wells sunk in the City. The Public Analyst has examined samples from these wells with apparently satisfactory results, and fifty-two buildings in the City are now relying on artesian wells in consequence. The popularity of the artesian well is, however, not yet so great as to lower appreciably the water-rest level of the water supplies already in existence. In the "Institutional Worker" of The Hospital only recently some very instructive comments were published concerning the use of artesian wells to hospitals in districts where the water-rate is excessive. In view of the above statement by the City Medical Officer, the recent suggestions in the "Institutional Worker" have added importance and interest.

### THE BRIGHTER SIDE OF THE MENTAL HOSPITAL.

It may be a matter of interest, in view of the article appearing in last week's issue of The Hospital on the "Brighter Side of the Mental Hospital," to note another element which can be made to figure largely in the happiness of the mental patient. A correspondent, with experience of a chaplain's duties in a mental hospital for women, points out that the religious side is one which appeals largely to the inmates, who grow exceedingly fond of their daily or weekly service, which seems to bring much happiness and comfort to them. It is not surprising that the part of such services which appears to appeal most to the patients is that of singing, and where the service, though of set form, is short and contains a large proportion of hymns, etc., it can be made to fill an important part in the life of the mental patient.

### THE LATEST ADDITION TO "DANGEROUS TRADES."

WE think it quite time that the occupation of clerk should be reckoned among unhealthy trades. Thanks to the latest report of the Medical Officer of Health for the City, the public conscience is being awakened to the evil which can be best summarised by the simple statement of fact that twice as many clerks die from diseases of the chest as do members of the general population. The National Union of Clerks is quoted as stating that 25 per cent. of the deaths among clerks are due to tuberculosis and 33⅓ per cent. to some form of lung disease. The occupation of clerk may be described as a dangerous trade for two reasons: first, that the employment itself is unhealthy, for man was not intended to be as sedentary as a vegetable, without the vegetable's compensating advantages of fresh air and sunlight; and, secondly, that the inevitable sedentariness of the work is aggravated by the bad conditions of ventilation which are prevalent in many, if not in most, offices. The City Medical Officer of Health refers to the Railway Office Bill, and says very pertinently that it is hard to understand why the provisions necessary for securing proper ventilation and lighting should be limited to railway offices. In 1912 Lord Salisbury introduced a Factory and Workshops (No. 2) Bill, which proposed that all underground workshops and offices should be brought under the jurisdiction of the Factories Acts. This would mean that the offices would be inspected and subject to the same penalties as workshops and factories, whereas now the Factory Inspector has frequently to enter a workshop through an office, where clerks are employed, but to which he is not allowed to apply the same tests and standard as to the workshop beyond. Nothing could well be more absurd, or more disastrous. It may come as a surprise to many that the despised clerk should be engaged on anything which can be called dangerous, but that is only because an insidious danger and a slow death have not the melodramatic attractions of a smash or a catastrophe. Yet, if melodramas are not to heighten our perception of the melodramatic, what are they for? The clerk is being sacrificed to the crudity which cannot grasp that one avoidable death is as tragic as another.

### AN OUTDOOR MEETING AT LEICESTER.

THE Leicester Saturday Hospital Society held its half-yearly meeting under perfect conditions in the grounds of the Society's new Convalescent Home for Women at Woodhouse Eaves. The delegates, some 300 in number, were conveyed from Leicester in brakes, and Sir Edward Wood presided over the proceedings. The committee's report stated that the number of beds in the Home had been raised to forty-five in order to meet the increasing applications for admission, and among staff changes we note that Miss Braye had been appointed matron of the Home in the place of Miss Oslar. At present trade conditions appeared to be good, and the National Insurance Act is stated in the report not to be affecting the workpeople's contributions. While 526 men and 348 women had been sent to convalescent homes during the half-year, an increase of 163 on last year's figures, it is not surprising that the work of the Leicester Royal Infirmary has greatly increased in the in-patients' department, and that there is a falling-off in the number of out-patients. The Children's Hospital reconstruction, we learn, is proceeding rapidly. The adoption of the report, which was moved from the chair, was seconded by Mr. S. A. Gunson and supported by Mr. J. Gipson Clarke and Mr. W. F. Hill, C.C., and carried. Afterwards the delegates enjoyed tea in the grounds, which, with the Home, were thrown open to the visitors.

### THE FINANCING OF CHARITY FETES.

THE result of the Noah's Ark Fair, which was opened on June 11 on behalf of the London Hospital, is that £1,900 has been handed over to that institution. The financing of charity fêtes is always worth scrutiny, and so we propose to examine the audited accounts in brief detail. The fête which was held in the Albert Hall, hired for the occasion at a sum of £757 odd, including the amounts paid for licence and the printing of tickets, involved a total expenditure of £2,107, of which about one-fourth was spent on decorations, while £344 and £320 were spent respectively on advertising and general expenses. Turning now to the receipts, we find that the total was £4,007 odd. Of this sum, premiums from the exhibition stalls realised £1,475, net receipts from these stalls £533, while the private stalls yielded £946. Tickets and collections brought in £553 and donations £326, while it is interesting to note that goods unsold at the fair, but otherwise bought, realised £83. We are afraid that the London Hospital must be somewhat disappointed at receiving £1,900 only out of £4,007 the total receipts. The hospital's authorities must have hoped for better things, for though £1,900 has been raised from a source which without expenditure and trouble would have yielded nothing, so much trouble and expenditure deserved a better return. The charity fête is still a somewhat speculative form of income-raising. It has not yet established itself as a "safe" investment, though in the region of charity finance safe investments are rare!

### A HOSPITAL FOUNDER'S JUBILEE.

IT is not often that a hospital founder lives to see the jubilee of his institution, yet this is the case with Mr. A. W. Mackenzie, who in 1863 assisted his brother, the late Sir Morell Mackenzie, to found the Throat Hospital in Golden Square. In taking advantage of the jubilee year to appeal for £12,000

to pay off the mortgage recently incurred by the purchase of 33 Golden Square, Mr. Mackenzie urges the claims of "the first institution for the special treament of throat diseases in London," and recalls the interest of the Empress Frederick in the institution for which she wrote a preface to the biographical sketch of her husband that Sir Rennell Rodd undertook at her request. So few hospital jubilees are the jubilees of their founders that Mr. Mackenzie's appeal must have a special force and a personal interest for all who are not in a position to understand the needs and work of his institution.

### COMPULSORY VACCINATION OF POLITICIANS.

Some three months or so ago an epidemic of smallpox began at Sydney, in which city the administration of vaccination laws had for a long time been notoriously lax. The outbreak is attributed to a case imported from Canada, and its nature was not at first suspected. The first batch of cases were mild in character, and were at first diagnosed as chicken-pox. As soon as the epidemic was detected the utmost resources of the sanitary service were at once put in action. Careful segregation, tracing of contacts, and such like measures were thoroughly carried out. Vaccination was also resorted to, and enormous numbers of the public rushed to their doctors to have this precautionary measure taken. At the present moment the epidemic is apparently still smouldering, if not actually widespread, for trouble has arisen with three itinerant British parliamentarians, who refused to undergo the compulsory vaccination ordained by the authorities for stamping out the disease, and were accordingly prohibited from disembarking from their ship. The gentlemen in question are Mr. W. Crooks, Mr. A. W. Black, and Mr. Edgar Jones. The first of the three is the well-known Labour member, who at least possesses common sense, however wild some may think his views to be on certain matters. Quite possibly it was his counsel which prevailed with the others, for at the moment of writing all three have submitted to the regulations, or at least announced an intention of doing so. We may possibly be wronging Mr. Black, but we judge by his political record that he is of exactly that curious type to whom "anti-fads" of all sorts strongly appeal. If so, the object-lesson presented to the three travellers by the epidemic in Sydney may be a most valuable experience. We congratulate them on their submission to the law, and we congratulate the authorities on their firmness in applying it without respect of persons.

### RESIDENTS' SALARIES AT COVENTRY HOSPITAL.

The house committee of the Coventry and Warwickshire Hospital, in its report for the four weeks ending August 13, states that: "In view of the extra work and responsibility arising from the recent growth of the institution, it was resolved to refer to a joint meeting of the medical and house committees the advisability of revising certain salaries." The joint meeting recommend: "That the senior house surgeon shall in future receive £150 per annum, rising to £160 at the end of his first year; that the house physician shall receive £110 per annum; and the junior house surgeon £100 per annum. The recommendation is made in the belief that this revision of salary will enable the hospital to secure the services of a senior officer who has held an appointment at a leading London hospital or at one of the large provincial hospitals, such as Liverpool or Manchester. This experience is regarded by the hon. medical staff as essential, but it is felt that if the applicant to the senior post shall have already held a resident appointment in this hospital such appointment shall be deemed sufficient qualification." Dr. Hawley, chairman of the medical committee, explained that residents were very difficult to secure, and that a man who had held hospital appointments starting at perhaps £80, and rising to £120, would not take another post at the same price. We are glad to quote this latest instance of the difficulty created by the scarcity of residents, and would draw the attention of Mr. E. W. Iliffe and Dr. Hawley to an article which we shall publish next week explaining the position, and outlining a policy for the hospitals to adopt concerning it. The title of the article will be "The Scarcity of Resident Medical Officers."

### THE CONTROL OF THE WORKHOUSE MASTER.

Whatever proposals may result in practical changes from the Draft Poor-Law Order, the sick wards, the lunatic wards, the nursery, and the maternity wards must be removed entirely from the control of the workhouse master. In a few cases where a separately administered infirmary is adjacent to the workhouse, the buildings are such that it would be possible and easy to transfer all these wards to the infirmary and separate them structurally from the workhouse. Wherever this is possible it should be done. In all other cases the medical officer of the workhouse should be made the medical superintendent of the wards in question. They should be entirely excluded from the administration of the workhouse master and matron. The master should occupy with respect to them the same position, and no more, as that of the steward of a separate infirmary. The subordinate staff of these wards should have no duties in the other wards of the workhouse and should be under the direct control of the medical officer, acting in the case of the female staff through the superintendent nurse, who would then become the matron of the sick wards. The engineering and labouring staff would remain under the general control of the master as is now the case in those Unions where this staff is common to both the workhouse and an adjacent and separate infirmary. Both the medical officer and the superintendent nurse should, of course, be directly responsible to the Board of Guardians or to a special committee of it, to whom they would report, and from whom they would receive instructions, just as is now the case in the separate infirmaries.

### A PIONEER SURGEON.

The medical profession in Manchester sustains a severe loss by the death of Mr. Walter Whitehead, who indeed occupied a distinct place in the development of surgery in England. After leaving the bleaching trade, to which family interests drew him, he became a medical student at the Chatham School of Medicine in Manchester, and filled up the interval before qualification by acting as locum to the residents in the Royal Infirmary there. Private practice in Nottingham occupied the next few years till, in 1867, he returned to Manchester to take up hospital appointments, both at St. Mary's Hospital for Women and Children and at the Royal Infirmary. It was not, however, with these or at the University, where he became Professor of Clinical Surgery and Examiner, that his fame became established, but in connection with Whitehead's operations for the removal of the tongue and of hæmorrhoids. That his methods were sufficient to make these two operations safe in the hands of those who were qualified to adopt them is only another way of saying that Whitehead was a surgical pioneer, and that his work was "bold." This reputation for boldness, however, attracted rather than repelled patients, who came to him from many distant places. He was a Fellow of the Royal Society, and his more important contributions to the literature of surgery include articles on the "Surgery of the Thyroid Gland" in Treves' "System" and on "Affections of the Mouth."

### REORGANISATION OF MEDICAL RELIEF IN BERMONDSEY.

Recently the Bermondsey Board of Guardians submitted to the Local Government Board a scheme for the complete reorganisation of their medical arrangements on the basis of a whole-time service. They suggested that the entire medical relief of the parish should be concentrated in the hands of the medical superintendent of the infirmary, who should be appointed medical officer of the two workhouses and of the five out-relief medical districts, in addition to his present post, at a salary of £550, rising to £650, with rations, furnished apartments, washing, and attendance; that the staff of assistant medical officers at the infirmary should be increased from two to five, and that they should act as deputies for the medical superintendent for the workhouses and out-relief districts. The first assistant medical officer was to receive £350, rising to £450, with rations, furnished apartments, etc., the second, third, and fourth assistant medical officers £450, rising to £500, without any emoluments, and the fifth £200, rising to £300, with the same emoluments as the first assistant. The reply of the Local Government Board to these suggestions has just been received. The Board states that any procedure in the direction of placing medical relief elsewhere than at the infirmary under the control of the medical superintendent should first be of a tentative and experimental nature. They would be prepared to approve an arrangement under which the medical superintendent should have responsibility for two of the out-relief districts with some addition to the infirmary staff, and in the event of the retirement of the medical officer of the two workhouses they would be willing that these institutions should also be placed under the medical superintendent's control. After considering this reply the Guardians decided to adhere to their scheme and appoint a deputation to wait on the Local Government Board.

### INFIRMARY SUPERINTENDENTS AND OUT-RELIEF.

As a matter of fact a similar scheme has been in force in Lambeth for some years past as regards some of the out-relief districts of that parish and has worked most successfully, so that the experimental stage may be said to be already passed. The advantages of the scheme are that it enables the cases most suitable for treatment in the infirmary to be selected by the medical superintendent, and that it enables a complete medical record of the patient to be kept and the after history to be followed up. Its disadvantage is that it throws so much administrative work upon the medical superintendent that he has very little time for clinical work.

### SIR JONATHAN HUTCHINSON'S WILL.

The will of Sir Jonathan Hutchinson, whose achievements and theories, especially in relation to leprosy, we summarised at the time of his death in June last, is now published. The gross value of his estate has been sworn at £92,269, of which, as perhaps will not surprise those who remember how much he was interested in the development of Hindhead, little more than one-third, £39,226, is net personalty. The three museums which he had established at Haslemere, Selby, and 22 Chenies Street he directed his trustees, but without imposing any trust, to dispose of according to his expressed wishes. He also directed that "the inscription on the gravestone is to be, in addition to the names and dates, 'A Man of Hope and Forward-Looking Mind.'" As no inscription interests the world more than an epitaph, this has been largely quoted in the lay press, and its simplicity is worthy of a scientific thinker.

### WORKHOUSE MEDICAL OFFICER AND SUPERINTENDENT NURSE.

The position of superintendent nurse under the new Draft Poor-Law Order is of the greatest importance to workhouse medical officers. Briefly summarised, the suggestions are that in every workhouse, having not less than 100 beds set apart for sick inmates, the Guardians shall appoint a superintendent nurse holding a certificate of at least three years' training from a recognised training school and being a midwife certified under the Midwives Act, 1902. The superintendent nurse so appointed is to supervise and control the nursing staff and the sick wards and any other wards that may be placed under her charge by the Guardians with the approval of the Local Government Board. She is to see that the inmates of these wards are properly attended to; that the directions of the medical officer, with respect to them, are carried out; and that her wards are properly cleansed, warmed, and ventilated. She is to have charge of the linen, bedding, and clothing in them, and is to report to the medical officer any defects and any matters requiring consideration in connection with her wards and with the nursing of the sick, and to the house committee, in a book to be submitted through the workhouse master, at every meeting of that committee upon all matters affecting her duties that may require consideration.

### WHERE THE ORDER NEEDS AMENDMENT.

From the explanations submitted with the Order by the Departmental Committee, they obviously contemplate the division of the administrative control (under the master and medical officer) between the two principal female officers—the superintendent nurse supervising and controlling the wards allotted to her and the matron the remaining wards for female inmates. But in the Order itself the matron has, as one of her duties, the supervision and control of the female officers. This will obviously need amendment if the expressed wish of the Departmental Committee to establish two principal female officers in these large workhouses is to be carried out. In workhouses having less than 100 beds set apart for sick inmates the Guardians are to appoint an officer to be called a head nurse, who is required to hold a certificate of three years' training from a recognised training school, or are to submit for the approval of the Local Government Board proposals whereby such skilled nursing attendance as may be likely to be required will be available. If a head nurse is appointed, and the matron does not possess the qualifications prescribed for that office, the head nurse shall in matters relating to the treatment and nursing of the sick act immediately under the directions of the medical officer.

### A TRAVELLING RADIOLOGIST FOR LONDON INFIRMARIES.

The Wandsworth Board of Guardians, at their last meeting, discussed a proposal submitted by Mr. D. Grundy that the Local Government Board should be asked to sanction an expenditure of at least £1,000 on the purchase of radium for St. John's Hill Infirmary. Mr. P. Rees thought the proper course would be to ask the authorities at Whitehall to place the whole of the radium treatment for the Metropolis in the hands of a central authority, such as the Asylums Board, who could appoint a travelling operator to visit the various infirmaries. Radium, he added, in the charge of an unqualified man might do more harm than good. Dr. Maccormac, the medical superintendent, informed the Board that there ought to be a sufficiently large amount of radium in the institution to ensure treatment for all cases. The Guardians, however, decided not to take action at present.

### MALINGERING: ITS CAUSE AND CURE.

A Special Committee of Inquiry has been appointed to inquire into malingering and the alleged excessive sickness claims on the part of insured persons. The Chairman of the Committee is Sir Claud Schuster, who is the legal member of the English Insurance Commission, and the other members include Miss Mona Wilson, the lady member of the Commission; Miss Mary Macarthur, secretary of the Women's Trade Union League; Mr. W. P. Wright, Grand Master of the Manchester Unity; Mr. A. H. Warren, president of the National Conference of Friendly Societies; Mr. Walter Davies, Sons of Temperance Society; the Chairman of the Manchester Insurance Committee; and three representatives of the medical profession. The Committee's terms of reference are "to inquire into and report upon the alleged excessive claims upon and allowances by approved societies in England in respect of sickness benefit and any special circumstances which may cause any such claims or allowances." The investigation is limited to England. Malingering, particularly among women, and the assertions that panel doctors have granted medical certificates to insured persons without proper examination and for trivial ailments will be specially inquired into. We may add, for those who are interested, that the extent to which panel doctors are liable to temptation in this respect will be discussed in a remarkable article which we hope to publish in the Special Educational Number of The Hospital that appears next week, entitled "The Prospects of Panel Practice."

### THE METROPOLITAN ASYLUMS BOARD AND SICK CHILDREN.

In a recent circular letter to the London Boards of Guardians the Metropolitan Asylums Board state that in future preference in admission to the Queen Mary's Hospital for Children at Carshalton and the Park Hospital for Children at Hither Green will be given to the undermentioned cases, which will be removed within a day or two of their notification to the offices of the Board—viz.: Non-pulmonary tuberculosis in its various forms; lateral curvature and other deformities, including children requiring Swedish exercises; paralysis; diseases of bones and joints not due to tubercle; and those requiring operation. A child who urgently required operation would be removed at once from its own home by the Board's ambulance. With regard to the first four categories it will be generally conceded that the equipment and position of the Board's hospitals for sick children are such as to justify Guardians in taking advantage of the Board's offer. The fifth category is, however, on quite a different footing. There is no more anxious time for a parent than that which precedes and follows the performance of an operation upon one of his or her children. Under such circumstances a parent would naturally wish to be in a position to see the child immediately before the operation and to know the result immediately after its performance, and it seems rather inhuman to suggest that at such a time a child should be removed to a hospital at a considerable distance from its home if such a separation can possibly be avoided. For this reason it appears to us that all operations should be performed at the local infirmary and that the children should not be transferred to the Board's hospitals until convalescent.

### DIAGNOSIS BY WIRELESS TELEGRAPHY.

The telephone consultation has long been a recognised part of medical practice, and grave debates have arisen concerning the exact rate of remuneration (if any) which a doctor should receive for advice thus rendered without seeing the patient, though the modern solicitor charges 3s. 6d. for answering the telephone. Obviously the telephone consultation is only legitimate when the doctor has already recently seen the patient, and when the latter or his nurse desires to notify the former of some fresh factor in the case which may demand an alteration of the course of treatment prescribed. A further stage in this form of " absent treatment " is reported from the South Pacific. According to the *Australasian Medical Gazette*, two steamers, the *Maheno* and the *Wimmera*, were on the sea between Australia and New Zealand. The former vessel received a " wireless " message from the latter asking if a doctor were on board. Receiving an affirmative answer, the *Wimmera* then sent a message to the effect that the captain was ill and gave some particulars of his symptoms. The doctor on the *Maheno* apparently succeeded in diagnosing the case on this information; at least he sent a prescription by return of wireless. Unfortunately the narrative stops at this interesting point, and no information is vouchsafed as to whether the captain recovered, or even whether he took the medicine prescribed.

### AN OPTICIAN AND MEDICAL PRACTICE.

As a result of an inquest on the body of a male child aged eight months, before Dr. T. Jackson, the Croydon Coroner, a verdict of " Death from natural causes " was returned, the jury refraining from adding a rider and from expressing an opinion. In the course of the evidence Horatio Franklin Sparling, who described himself as an optician, produced the prescription which he had handed to the father of the deceased child, and the Coroner remarked that it was one which no practitioner would regard as improper for the case, the symptoms of which were diarrhœa and sickness. Sparling, who was described as having a brass plate outside his door and a red lamp, besides a window on which hours of attendance were painted, said that since the baby's death the words on the brass plate had been changed to " H. F. Sparling " (the prefix " Dr." apparently being deleted), and that the word " Surgery " on the lamp had over it the representation of a pair of spectacles. He added that " this paraphernalia " was the record of a fully qualified medical man who had been in practice at the house, and who was in partnership with himself, though there was no written agreement. The witness, after pressing, added also that his former partner was the medical man to whom he had referred the child's father when the father had sent for him on the morning after he had given the prescription. Dr. James Morrison Orr, in the course of his evidence, said his former partnership with Sparling was more in the nature of a friendship than an actual partnership. Mr. Sparling used to dispense, under his supervision, in the surgery. The Coroner thereupon remarked that the General Medical Council had struck many men from the Register for having unqualified assistants, and after the jury had given their verdict he addressed the two witnesses referred to and said that both had sailed very near the wind, and that he hoped that Mr. Sparling would take warning and not carry on what was really a deception on the public—a summary with which we cordially agree.

### DISPENSERS WANTED.

Pharmacists are complaining of the difficulty they experience in finding assistants—a difficulty which seems to be becoming more pronounced. This is, of course, due to the National Insurance Act, the effect of which has been to transfer the work of dispensing for a large proportion of the community from the doctors to the chemists. Dispensers who have had experience with doctors or in public institutions should find little difficulty in obtaining posts as dispensers to pharmacists at salaries above, rather than below, those which they were formerly paid. It may be well to point out that any dispenser who for three years immediately prior to the passing of the Insurance Act acted as a dispenser to a duly qualified medical practitioner or a public institution is eligible to dispense medicines for a panel chemist without actually being under the supervision of the chemist, and is therefore in a position to expect to be well paid for his services. But a dispenser without the three years' qualification can also dispense for insured persons if the work is done under qualified supervision, and there are now many openings in chemists' shops which such dispensers could fill.

### THIS WEEK'S DRUG MARKET.

Business in the Drug market continues quiet, but not unusually so for the time of year, and the tone is by no means unhealthy. Of price changes there have been few of any importance during the week. There has been a slight advance in opium prices, but possibly this is only temporary; in any case, cautious buyers do not seem to be in a hurry to come on to the market. There has been a steady demand for morphine, the price of which is unchanged. Cream of tartar is rather dearer, and in good demand. Citric acid continues scarce, and the price is well maintained. A fair business has been done in Belgian chamomiles, which are now coming on to the market. At the recent public sale of drugs in Mincing Lane Cape aloes sold at cheaper rates; cardamoms were rather dearer; ipecacuanha fetched higher prices; Jamaica sarsaparilla was slightly cheaper. A steady business continues to be done in quinine, and an advance in price is likely.

